# The Optimal Cut-Off Point for Thai Diagnostic Autism Scale and Probability Prediction of Autism Spectrum Disorder Diagnosis in Suspected Children

**DOI:** 10.3390/healthcare10101868

**Published:** 2022-09-25

**Authors:** Duangkamol Tangviriyapaiboon, Suttipong Kawilapat, Samai Sirithongthaworn, Hataichanok Apikomonkon, Chidawan Suyakong, Pimwarat Srikummoon, Salinee Thumronglaohapun, Patrinee Traisathit

**Affiliations:** 1 Rajanagarindra Institute of Child Development, Chiang Mai 50180, Thailand; 2 Department of Statistics, Faculty of Science, Chiang Mai University, Chiang Mai 50200, Thailand; 3 Department of Mental Health, Ministry of Public Health, Nonthaburi 11000, Thailand; 4 Department of Occupational Therapy, Faculty of Associated Medical Sciences, Chiang Mai University, Chiang Mai 50200, Thailand; 5 Data Science Research Center, Department of Statistics, Faculty of Science, Chiang Mai University, Chiang Mai 50200, Thailand; 6 Research Center in Bioresources for Agriculture, Industry and Medicine, Department of Statistics, Faculty of Science, Chiang Mai University, Chiang Mai 50200, Thailand

**Keywords:** cut-off point, diagnosis, autism spectrum disorder, Thai Diagnostic Autism Scale, TDAS

## Abstract

The Thai Diagnostic Autism Scale (TDAS) was developed to diagnose autism spectrum disorder (ASD) under the context and characteristics of the Thai population. Although the tool has an excellent agreement, the interpretation of diagnostic results needs to rely on the optimal cut-off point to maximize efficiency and clarity. This study aims to find an optimal cut-off point for TDAS in the diagnosis of ASD and to compare its agreement with the DSM-5 ASD criteria. This study was conducted on 156 children aged 12–48 months old who were suspected of having ASD and had enrolled from hospitals in the four regions of Thailand in 2017–2018. The optimal cut-off point for TDAS was considered by using receiver operating characteristic (ROC) curves according to the DSM-5 ASD criteria. The areas under the curve (AUCs) for TDAS and ADOS-2 were also compared. Multivariable logistic regression was performed to create a predictive model for the probability of ASD. The AUC of TDAS was significantly higher than that of ADOS-2 (0.8748 vs. 0.7993; *p* = 0.033). The optimal cut-off point for TDAS was ≥20 points (accuracy = 82.05%, sensitivity = 82.86%, and specificity = 80.93%). Our findings show that TDAS with a cut-off point can yield higher diagnostic accuracy than ADOS-2 and TDAS domain. Diagnosis by using this cut-off point could be useful in practical assessments.

## 1. Introduction

Autism spectrum disorder (ASD) is a complex developmental condition characterized by persistent deficits in social communication and interaction across multiple contexts coupled with restricted and repetitive patterns of behavior, interests, or activities [1]. In recent decades, ASD has become a major public health problem with a worldwide prevalence of approximately 1 in 100 children [2]. Studies carried out during the last two decades indicate that North America and Europe have the highest prevalence of ASD, with median rates of 0.90% and 0.61%, respectively, while the incidence rate in Thailand in children aged less than 12 years old has increased from 1.43 per 10,000 in 1998 to 6.94 per 10,000 in 2002 [3,4]. More recently, the prevalence of ASD is approximately 1 in 161 children aged less than 5 years old in Thailand, and the number of new cases is likely to continue increasing [5]. The early detection of ASD facilitates early intervention, which can help to substantially reduce lifetime healthcare costs.

Several well-known and widely used standardized assessment tools are used for the detection of ASD, such as the Autism Diagnostic Observation Schedule [6,7], the Autism Diagnostic Interview-Revised (ADI-R) [8,9], and the Childhood Autism Rating Scale (CARS) [10,11]. In a previous systematic review, Randall et al. [12] concluded that the sensitivity and specificity of ADOS ranged from 0.76 to 0.98 and from 0.20 to 1.00; that those of CARS ranged from 0.66 to 0.89 and from 0.21 to 1.00; and that those of ADI-R ranged from 0.19 to 0.75 and from 0.63 to 1.00, respectively. They also compared the summary statistics for ADOS, CARS, and ADI-R and found that ADOS was the most sensitive while all tools performed similarly for specificity.

The identification of ASD in Thailand has been widely performed by specialist physicians in the fields of child and adolescent psychiatry, pediatrics, and general psychiatry by using standard diagnostic tools such as the Autism Diagnostic Observation Schedule, second edition (ADOS-2) [7,13], which currently comprises the ASD diagnostic criteria for the International Classification of Diseases (ICD) or the fifth edition of Diagnostic and Statistical Manual of Mental Disorders (DSM-5) [14,15]. Assessment tools previously developed to detect ASD and other Pervasive Developmental Disorders (PDDs) in early childhood in Thailand include the PDD Screening Questionnaire for children aged 1–4 years old (PDDSQ 1–4) [16] and the short version of the PDDSQ for children aged 1–5 years old (PDDSQ-short version) [17]. However, a valid criticism about the comprehensiveness of these tools is that evaluation is based only on parental observations, which could lead to misleading evaluations. Differences in cultural factors when translating a western diagnosis tool might influence the validity and reliability of the instruments [18]. In addition, another barrier is the cost of certification training for western-standardized tools; it is quite high and not covered by the healthcare system in Thailand [19]. Meanwhile, the limited number of current practitioners will inevitably have led to missed or late diagnoses of ASD. Therefore, the Thai Diagnostic Autism Scale (TDAS) was recently developed as a new tool primarily used for the early diagnosis of ASD among Thai children aged 12–48 months old [20]. TDAS is designed to be used by multidisciplinary teams comprising health professionals and nurses but can also serve as an additional ASD diagnostic instrument for physicians. This tool can be used to comprehensively identify the symptoms related to ASD that are only briefly covered in DSM-5 and presents clearer criteria for their interpretation within the Thai cultural context.

TDAS consists of 23 items (13 and 17 items in the observational and interview sections, respectively) classified into seven domains (A1: deficits in social-emotional reciprocity; A2: deficits in non-verbal communicative behavior used for social interaction; A3: deficits in developing, maintaining, and understanding relationships; B1: stereotyped or repetitive motor movements, use of objects, or speech; B2: sameness, inflexible adherence to routines, or ritualized pattern; B3: highly restricted fixed interests that are abnormal in intensity or focus; and B4: hyper- or hypo-reactivity to sensory input or unusual interest in sensory aspects of the environment) according to the DSM-5 ASD criteria. These were assessed via an observation conducted during an examiner/child interaction with a parent present in a private room adjusted for the child’s comfort and via an interview with the parent(s). Six items were scored in both the observation and interview sections, with seven strictly as observation and the remaining ten as interview only. Stereotyped or repetitive motor movements, the use of objects, and/or speech (B1) were scored by both the examiner after observation and in the interview with parent(s), whereas deficits in communicative behavior used for social interaction (A2) were scored after only observation and drawing on the expertise of the examiner related to this area. The interview with parent(s) was determined as the best method to score sameness, inflexible adherence to routines, and/or ritualized patterns (B2). TDAS showed good overall content validity (IOC range 0.71–1.00), suitable construct validity (root-mean-squared errors of approximation of 0.076 and 0.067, comparative fit indexes of 0.902 and 0.858, and Tucker–Lewis indexes of 0.882 and 0.837 for the observation and interview sections, respectively), and excellent diagnostic agreement with evaluators (Kappa = 1.000) and physicians’ diagnoses (Kappa = 0.871). The sensitivity and specificity of TDAS were 100% and 82.4%, respectively. Children with a single score for each of the A1–A3 domains and at least two of the B1–B4 domains were classified as having ASD [20].

Even though the criteria for ASD diagnosis based on the TDAS domains were in excellent agreement with the physicians’ diagnoses, interpretation of the results can be challenging in practice. Therefore, the aim of this pilot study is to find an optimal cut-off point for TDAS and to compare its efficacy with the TDAS domain criteria and ADOS-2 according to the DSM-5 ASD criteria.

## 2. Materials and Methods

### 2.1. Participants and Settings

The participants in this study were children suspected of having ASD aged 12–48 months old who enrolled through 9 secondary and tertiary hospitals in 4 regions of Thailand (northern region: the Nakornping Hospital, the Chomthong Hospital, the Lamphun Hospital, the Lampang Hospital, the Phayao Hospital, and the Chiangrai Prachanukroh Hospital; northeastern region: the Khon Kaen Hospital; central region: the Bang Phli Hospital; and southern region: the Surat Thani Hospital) in 2017–2018. The required sample size was calculated by using Buderer’s formula [21] as follows:

(1) $$n=\left[Z_{1-\alpha /2}^{2}\cdot S_{p}{(1-S}_{p})\right]{/(L}^{2}\cdot (1-P))\;\;=\left[\left(1.96^{2}\right)\left(0.90\right)\left(1-0.90\right)\right]/\left(\left(0.05^{2}\right)\left(1-0.006\right)\right)=139.1\approx 140,$$

where *n* is the required sample size, *Z*_1−*α*/2_ is the statistic corresponding to the level of confidence (*Z*_0.975_ = 1.96), *S_p_* is the anticipated specificity (assumed as 0.90 in this study), *P* is the expected prevalence of ASD (assumed as 0.006 (1 in 161 children) according to the previously reported prevalence of ASD [5]), and *L* is the absolute precision (assumed as 0.05 in this study).

### 2.2. Assessment

The clinical diagnosis of ASD in the children (ASD or non-ASD) was based on the DSM-5 criteria. The classification of ASD using ADOS-2 and the TDAS domain criteria to compare with the proposed cut-off point was made according to the ADOS-2 scoring guidelines and the TDAS domain criteria guidelines, respectively. Scoring and interpretation were performed after the diagnosis according to DSM-5 criteria by three physicians: one conducted a face-to-face interview, while the other two reviewed the video recording and the child’s medical record.

ADOS-2 consists of 5 modules, including Modules 1–4 and the Toddler Module [7]. The administration of each module generally took 40–60 min, with an additional 30–45 min for scoring and interpretation. Choosing which module to evaluate ASD was dependent on the chronological age and expressive language development of the child. The children would attend several activities, and their behavior would be observed and recorded. Assessment of the Thai language version of ADOS-2 was conducted by authorized examiners with clinical or research certification training who met the criterion of 80% agreement with the research reliable trainers and experience in assessing ASD in their daily work. The ADOS-2 examiners in this study included physicians, psychiatrists, nurses, clinical psychologists, occupational therapists, and speech pathologists.

The TDAS items were consisted of 7 domains: (A1) deficits in social-emotional reciprocity; (A2) deficits in non-verbal communicative behavior used for social interaction; (A3) deficits in developing, maintaining, and understanding relationships; (B1) stereotyped or repetitive motor movements, use of objects, or speech; (B2) sameness, inflexible adherence to routines, or ritualized pattern; (B3) highly restricted fixed interests that are abnormal in intensity or focus; and (B4) hyper- or hypo-reactivity to sensory input or unusual interest in sensory aspects of the environment. The administration of TDAS generally took 40 min for evaluation and 20–30 min for scoring. The score of each item ranges from 0 (none or rarely) to 2 (often or nearly every time). Thus, the plausible scores from TDAS ranged from 0 to 60 points. Children were classified as ASD if they attained a single score for each of the A1–A3 domains and at least two of the B1–B4 domains [20]. The TDAS assessment was conducted by hospital staff with clinical or research certification after undergoing 3 days of training for TDAS administration. All of the participants attended both the lecture and workshop sections. The TDAS examiners who met the research reliability criterion of 80% with the research certified trainers were certificated and could reliably use the TDAS for the diagnosis of ASD in children.

The authors of the diagnostic tools, ADOS-2, used in this study gave permission for their use, and appropriate licenses were issued to the Principal Investigator, Duangkamol Tangviriyapaiboon, M.D., by the publisher (Western Psychological Services, CA, USA).

### 2.3. Statistical Analysis

Descriptive statistics of the characteristics of the children are presented as frequencies and percentages for categorical variables, and median and interquartile ranges for continuous variables.

The optimal cut-off point for ASD diagnosis using TDAS according to the DSM-5 criteria was obtained by using receiver operating characteristic (ROC) area under the curves (AUCs), as suggested by Liu [22]. The cut-off point that maximized the concordance probability (the objective function of sensitivity and specificity) was considered optimal. The ROC AUCs for TDAS and the gold standard, ADOS-2, were compared by using the *roccomp* test package in Stata [23], an algorithm for comparing the estimated covariance matrices of the ROC AUCs via a Chi-squared test [24]. Sensitivity and specificity were also calculated as indices for correct classification of ASD according to the cut-off point [25,26]. Agreement between the ADOS-2 and TDAS scores on the DSM-5 ASD criteria was examined using a kappa agreement comparison [27]. These analyses were considered only for the participants with completed data of TDAS and ADOS-2 measurement.

TDAS score, gender, and age were included in a multivariable logistic regression model with backward elimination to create a predictive model for the probability of ASD. Nagelkerke’s *r*^2^ was used to explain variation in the predictive model for the diagnosis of ASD according to the DSM-5 ASD criteria [28,29].

All statistical significances were considered as *p* < 0.05. All analyses were performed using Stata 17.

### 2.4. Ethical Approval and Consent for Participation

Ethical approval for this study was obtained from the RICD ethics committee (number 14/2015 and 1/2017). Written informed consent by the parents of the participants was given prior to participation in the study.

## 3. Results

### 3.1. The Characteristics of the Participants

Of the 156 children who participated in this study, 124 (80%) were male and the median age was 34 months old (interquartile range (IQR) = 27–41). The proportion of boys was higher in the autism group (86.5% vs. 66.7%, *p* = 0.005), and the age of the children with autism was higher (36 (31–41) vs. 28 (24–40) months). The median scores for ADOS-2 were 14 (10–20) and 22 (18–25) for the non-ASD and ASD groups, respectively (*p* < 0.001). Similarly, the median scores for TDAS were 11 (8–17) and 26 (17–33) for the non-ASD and ASD groups, respectively (*p* < 0.001) (Table 1).

### 3.2. The Optimal Cut-Off Point for TDAS According to the DSM-5 ASD Criteria

**Figure 1:**
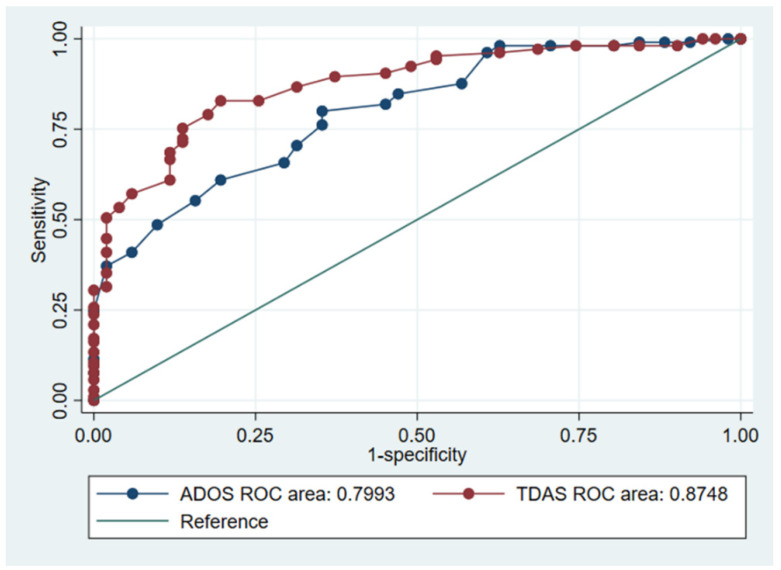
ROC curves for the ADOS-2 and TDAS scores according to the DSM-5 ASD criteria.

The results from the univariable logistic regression analysis show that both the ADOS-2 (odds ratio (OR) = 1.22; 95% confidence interval (CI) = 1.14–1.32; *p* < 0.001) and TDAS scores (OR = 1.20; 95% CI = 1.13–1.27; *p* < 0.001) are positively associated with the DSM-5 ASD criteria. Meanwhile, the ROC AUC of TDAS was significantly higher than that of ADOS-2 (0.8748 vs. 0.7993; *χ*^2^ = 4.54; *p* = 0.033) (Figure 1 and Table 2). According to Liu’s method, the optimal cut-off point for TDAS was ≥20 points, which yielded the highest diagnosis accuracy (82.05%), as well as high sensitivity (82.86%) and specificity (80.93%) (Table 3).

### 3.3. ASD Diagnosis Comparison between ADOS-2, TDAS, and TDAS ≥20 Points

According to the results of the Kappa test, the autism diagnoses using the three methods are in significant agreement with the DSM-5 ASD criteria, with that of TDAS (≥20 points) being the highest (82.05%), followed by the TDAS domains criteria (75.64%), and ADOS-2 (72.44%) (Table 4).

### 3.4. The Predictive Model for the Probability of ASD

According to multivariable logistic regression analysis, TDAS score (coefficient = 0.179, 95% confidence interval (CI) = 0.112–0.240, *p* < 0.001) and age (coefficient = 0.068, 95% CI = 0.014–0.122, *p* = 0.013) were significantly associated with ASD (Table 5). A formula to predict the probability of ASD based to these variables was defined as follows:

(2) $${\;\mathrm{Pr}(\mathrm{ASD})=e}^{-5.341+0.179(\mathrm{TDAS})+0.068\left(\mathrm{Age}\right)}/\left({1+e}^{-5.341+0.179(\mathrm{TDAS})+0.068\left(\mathrm{Age}\right)}\right),$$

where Pr(ASD) is the probability of ASD, TDAS is the TDAS score (ranging from 0 to 60), and Age is the age of the child in months. For example, the probability of ASD is predicted as 0.665 if a child aged 36 months old has a TDAS score of 20. Examples of the predicted probabilities for a child aged 12, 24, 36, or 48 months old are presented in Table S1.

## 4. Discussion

This preliminary study was conducted on children suspected of having ASD who had enrolled at one of the hospitals in Chiang Mai, Thailand, to find an optimal cut-off point for the diagnosis of ASD using TDAS. Our findings show that TDAS ≥20 points yielded high accuracy (82.05%), sensitivity (82.86%), and specificity (80.39%) for ASD diagnosis. Compared to a recent study to find the optimal cut-off point for ADOS-2 in toddlers aged 12–30 months old in the US, the cut-off points were 12 and 10 based on Algorithms 1 and 2, respectively (Algorithm 1 for toddlers aged 12–20 months old or 21–30 months with <5 words and Algorithm 2 for toddlers aged 21–30 months old with ≥5 words) [30]. The sensitivity values were 98.31% and 95.65%, the specificity values were 75.41% and 84.78%, and the accuracy values were 93.60 and 91.30 for Algorithms 1 and 2, respectively. Although the accuracy and sensitivity in our study were lower than these, the specificity is similar for both age groups.

Compared to other assessment tools for ASD in Thailand, the sensitivity of TDAS in our study was similar (82%) but specificity was lower (94%) than those for PPDSQ 1–4 [16], whereas its sensitivity was higher (70.8%) but specificity was lower (93.6%) than those for the PPDSQ-short version [17]. The differences in sensitivity and specificity could be related to the outcomes of the previous studies being considered for all PDDs in children, whereas ours was focused on ASD only.

The findings from a previous study in China show that, whereas the Modified Checklist for Autism in Toddlers (M-CHAT) yielded high sensitivity (88.3%) but low specificity (36%) and the Autism Behavior Checklist (ABC) yielded low sensitivity (27.2%) but high specificity (87.3%), combining them provided a good trade-off between sensitivity (85.5%) and specificity (56.6%) [31]. Similar to this scenario, adequately high sensitivity and specificity presented in our study might have resulted from the review and selection of key items from the existing ASD assessment tools during the development of TDAS. Moreover, considering both observational and interview sections might also have resulted in better sensitivity and specificity than using only one aspect. Nevertheless, the larger set of items may make using TDAS take too long in practice.

Both the TDAS and ADOS-2 scores are positively associated with ASD diagnosis based on the DSM-5 ASD criteria. However, when considering the ROC AUCs, that of the TDAS domain criteria was slightly higher than that of the ADOS-2 score, which could be because TDAS was developed and adapted for the Thai context and society, whereas barriers related to the raters or cultural differences may be prevalent in the ADOS-2 items. It is noteworthy that cultural factors were not addressed for the gold standard assessment tools for diagnosing ASD (ADOS and the Autism Diagnostic Interview), which might have influenced the validity and reliability of these instruments [18]. Even though its accuracy was not vastly higher than that of ADOS-2, using the TDAS domain criteria to diagnose ASD could advantageously decrease the effect of cultural influence. In addition, we also showed that using the optimal cut-off point of ≥20 increased the diagnosis accuracy of TDAS. Moreover, another barrier is the cost of certification training for western-standardized tools; it is quite high and not covered by the healthcare system in Thailand [19]. Hopefully, increasing the accessibility of TDAS to practitioners will reduce the number of children with missed or late diagnoses of ASD.

In addition, ASD diagnosis using the provided cut-off point for TDAS was more accurate than using the domain criteria, which could be because the TDAS domain criteria are only focused on the occurrence of ASD-related behavior according to each item whereas TDAS with the cut-off point considers the frequency of each behavior. Nevertheless, considering the frequency or severity of some behaviors may bias the evaluation, especially the interview section for the parental view. However, targeted training of evaluators should reduce the effect of bias.

Other than the TDAS score, our findings show that the age of the children was independently associated with ASD. As shown in a recent study in the US, cut-off points for toddlers were different between each age group [30]. However, the researchers did not attempt to find the optimal cut-off point for the subgroups due to the limited number of children in each age range. Therefore, determining optimal cut-off points for specific age groups would be of interest in a future study.

Our study also provided a predictive model for the probability of ASD based on the TDAS score adjusted for the ages of the children based on Nagelkerke’s *r*^2^. The variation in the probability of ASD of 52.9% might be related to the limited number of variables available (TDAS score, gender, and age). Even though the level of explained variation was not high, consideration of the TDAS score and age-adjusted probability prediction could be useful for decision-making on the prevention and treatment of ASD in practice.

The main limitation of this study is that the range of participants might not be representative of all children suspected of having ASD nationwide because most of the children were only recruited in the hospitals in the northern region. Another limitation is the limited number of variables included in the predictive model. A nationwide study with more variables in the predictive model concerning other potentially associated factors of ASD such as parental age [32,33] should be conducted.

## 5. Conclusions

The development and validation of tools for evaluating and diagnosing ASD in different languages and cultural contexts is a hot topic. Our findings show that TDAS with a cut-off point had a higher accuracy of ASD diagnosis than ADOS-2 and the TDAS domain criteria. As well as being useful for the practical assessment of ASD in children. this tool could help to reduce barriers due to cultural differences and the cost of training using Western or translated assessment tools, thereby increasing its accessibility in Thailand. This will hopefully reduce the number of children with missed or late diagnoses of ASD. Further study involving subgroup analysis with a larger group of participants nationwide and including other potential ASD-associated factors should be conducted.

## Figures and Tables

**Table 1:** Characteristics of the participants (*N* = 156).

Characteristic (*n* (%) or Median (IQR))	All	DSM-5		
		Non-ASD (*n* = 51)	ASD (*n* =105)	*p*-Value ^a^
Gender (n = 155)				0.005
Boys	124 (80.0%)	34 (66.7%)	90 (86.5%)	
Girls	21 (20.0%)	17 (33.3%)	14 (13.5%)	
Age (months) (n = 152)	34 (27–41)	28 (24–40)	36 (31–41)	0.001
ADOS-2 score	20 (14–25)	14 (10–20)	22 (18–25)	<0.001
TDAS score	25 (16–32)	14 (9–19)	30 (22–26)	<0.001

^a^*p*-value derived by using a Fisher’s exact test for categorical variables and a Mann–Whitney U test for continuous variables. n, frequency; %, percentages; IQR, interquartile range; ADOS-2, the Autism Diagnostic Observation Schedule-Second Edition; TDAS, the Thai Diagnostic Autism Scale.

**Table 2:** Comparison of the ROC AUCs of the ADOS-2 and TDAS scores according to the DSM-5 ASD criteria (N = 156).

Tools	ROC AUC	SE	95% CI	*p*-Value ^a^
ADOS-2 (gold standard) ^b^	0.7993	0.0359	(0.7289–0.8698)	
TDAS ^b^	0.8748	0.0283	(0.8193–0.9303)	0.033

^a^*p*-value derived by using roccomp test based on the algorithm of DeLong, DeLong, and Clarke-Pearson (1988). ^b^ Significant association with the DSM-5 ASD criteria (*p* < 0.001). ROC, receiver operating characteristic; AUC, area under the curve; SE, standard error; CI, confidence interval; ADOS-2, the Autism Diagnostic Observation Schedule-Second Edition; TDAS, the Thai Diagnostic Autism Scale.

**Table 3:** Characteristics of the cut-off points for TDAS according to the DSM-5 ASD criteria.

Cut-Off Point	Sensitivity	Specificity	Accuracy
≥2	100.00%	0.00%	67.31%
≥4	100.00%	3.92%	68.59%
≥6	100.00%	5.88%	69.23%
≥7	98.10%	9.80%	69.23%
≥8	98.10%	15.69%	71.15%
≥9	98.10%	19.61%	72.44%
≥10	98.10%	25.49%	74.36%
≥11	97.14%	31.37%	75.64%
≥12	96.19%	37.25%	76.92%
≥13	95.24%	47.06%	79.49%
≥14	94.29%	47.06%	78.85%
≥15	92.38%	50.98%	78.85%
≥16	90.48%	54.90%	78.85%
≥17	89.52%	62.75%	80.77%
≥18	86.67%	68.63%	80.77%
≥19	82.86%	74.51%	80.13%
≥20	82.86%	80.39%	82.05%
≥21	79.05%	82.35%	80.13%
≥22	75.24%	86.27%	78.85%
≥23	72.38%	86.27%	76.92%
≥24	71.43%	86.27%	76.28%
≥25	68.57%	88.24%	75.00%
≥26	66.67%	88.24%	73.72%
≥27	60.95%	88.24%	69.87%
≥28	57.14%	94.12%	69.23%
≥29	53.33%	96.08%	67.31%
≥30	50.48%	98.04%	66.03%
≥31	44.76%	98.04%	62.18%
≥32	40.95%	98.04%	59.62%
≥33	35.24%	98.04%	55.77%
≥34	31.43%	98.04%	53.21%
≥35	30.48%	100.00%	53.21%
≥36	25.71%	100.00%	50.00%
≥37	23.81%	100.00%	48.72%
≥38	20.95%	100.00%	46.79%
≥39	17.14%	100.00%	44.23%
≥40	16.19%	100.00%	43.59%
≥41	13.33%	100.00%	41.67%
≥42	10.48%	100.00%	39.74%
≥43	9.52%	100.00%	39.10%
≥44	7.62%	100.00%	37.82%
≥45	5.71%	100.00%	36.54%
≥46	2.86%	100.00%	34.62%
≥48	0.95%	100.00%	33.33%
>48	0.00%	100.00%	32.69%

**Table 4:** ASD diagnosis comparison according to the DSM-5 ASD criteria.

Criteria	DSM-5			
	Non-ASD (n = 51)	ASD (n = 105)	Agreement	*p*-Value ^a^
ADOS-2			113 (72.44%)	<0.001
Non-ASD	9 (17.65%)	1 (0.95%)		
ASD	42 (82.35%)	104 (99.05%)		
TDAS ^b^			118 (75.64%)	<0.001
Non-ASD	22 (43.14%)	9 (8.57%)		
ASD	29 (56.86%)	96 (91.43%)		
TDAS ≥ 20 points			128 (82.05%)	<0.001
Non-ASD	41 (80.39%)	18 (17.14%)		
ASD	10 (19.61%)	87 (82.86%)		

^a^*p*-value derived from kappa agreement comparison. ^b^ TDAS diagnosis of autism considered based on the condition three domains of section A and at least two domains of section B.

**Table 5:** Factors associated with ASD according to the DSM-5 ASD criteria (N = 152).

Variables	Coefficient (95% CI)	*p*-Value ^a^	Nagelkerke *r*^2^
Constant	−5.341 (−7.678, −3.003)	<0.001	0.529
TDAS score	0.179 (0.119, 0.240)	<0.001	
Age (months)	0.068 (0.014, 0.122)	0.013	

^a^*p*-value derived from multivariable logistic regression. CI, confidence interval; TDAS, the Thai Diagnostic Autism Scale.

## Data Availability

The datasets used and/or analyzed during the current study are available from the corresponding author upon reasonable request.

## Supplementary Materials

The following supporting information can be downloaded at https://www.mdpi.com/article/10.3390/healthcare10101868/s1, Table S1: The predicted probability of ASD based on TDAS score and age.
