# Supplementary material for: The Optimal Cut-Off Point for Thai Diagnostic Autism Scale and Probability Prediction of Autism Spectrum Disorder Diagnosis in Suspected Children
Source: Healthcare (Basel). 2022 Sep 25;10(10):1868. doi: 10.3390/healthcare10101868 (PMC9602004; doi:10.3390/healthcare10101868)
Supplement: Supplementary file 1 [file healthcare-10-01868-s001.zip › healthcare-1901502-supplementary.pdf]

Table S1. The predicted probability of ASD based on TDAS score and age.

Age (months)	TDAS Score	Z	Predicted Probability	TDAS Score	Z	Predicted Probability	TDAS Score	Z	Predicted Probability
**12**	**0**	-4.525	0.011						
	**1**	-4.346	0.013	**21**	-0.766	0.317	**41**	2.814	0.943
	**2**	-4.167	0.015	**22**	-0.587	0.357	**42**	2.993	0.952
	**3**	-3.988	0.018	**23**	-0.408	0.399	**43**	3.172	0.960
	**4**	-3.809	0.022	**24**	-0.229	0.443	**44**	3.351	0.966
	**5**	-3.630	0.026	**25**	-0.050	0.488	**45**	3.530	0.972
	**6**	-3.451	0.031	**26**	0.129	0.532	**46**	3.709	0.976
	**7**	-3.272	0.037	**27**	0.308	0.576	**47**	3.888	0.980
	**8**	-3.093	0.043	**28**	0.487	0.619	**48**	4.067	0.983
	**9**	-2.914	0.051	**29**	0.666	0.661	**49**	4.246	0.986
	**10**	-2.735	0.061	**30**	0.845	0.700	**50**	4.425	0.988
	**11**	-2.556	0.072	**31**	1.024	0.736	**51**	4.604	0.990
	**12**	-2.377	0.085	**32**	1.203	0.769	**52**	4.783	0.992
	**13**	-2.198	0.100	**33**	1.382	0.799	**53**	4.962	0.993
	**14**	-2.019	0.117	**34**	1.561	0.826	**54**	5.141	0.994
	**15**	-1.840	0.137	**35**	1.740	0.851	**55**	5.320	0.995
	**16**	-1.661	0.160	**36**	1.919	0.872	**56**	5.499	0.996
	**17**	-1.482	0.185	**37**	2.098	0.891	**57**	5.678	0.997
	**18**	-1.303	0.214	**38**	2.277	0.907	**58**	5.857	0.997
	**19**	-1.124	0.245	**39**	2.456	0.921	**59**	6.036	0.998
	**20**	-0.945	0.280	**40**	2.635	0.933	**60**	6.215	0.998
**24**	**0**	-3.709	0.024						
	**1**	-3.530	0.028	**21**	0.050	0.512	**41**	3.630	0.974
	**2**	-3.351	0.034	**22**	0.229	0.557	**42**	3.809	0.978
	**3**	-3.172	0.040	**23**	0.408	0.601	**43**	3.988	0.982
	**4**	-2.993	0.048	**24**	0.587	0.643	**44**	4.167	0.985

Age (months)	TDAS Score	Z	Predicted Probability	TDAS Score	Z	Predicted Probability	TDAS Score	Z	Predicted Probability
	**5**	-2.814	0.057	**25**	0.766	0.683	**45**	4.346	0.987
	**6**	-2.635	0.067	**26**	0.945	0.720	**46**	4.525	0.989
	**7**	-2.456	0.079	**27**	1.124	0.755	**47**	4.704	0.991
	**8**	-2.277	0.093	**28**	1.303	0.786	**48**	4.883	0.992
	**9**	-2.098	0.109	**29**	1.482	0.815	**49**	5.062	0.994
	**10**	-1.919	0.128	**30**	1.661	0.840	**50**	5.241	0.995
	**11**	-1.740	0.149	**31**	1.840	0.863	**51**	5.420	0.996
	**12**	-1.561	0.174	**32**	2.019	0.883	**52**	5.599	0.996
	**13**	-1.382	0.201	**33**	2.198	0.900	**53**	5.778	0.997
	**14**	-1.203	0.231	**34**	2.377	0.915	**54**	5.957	0.997
	**15**	-1.024	0.264	**35**	2.556	0.928	**55**	6.136	0.998
	**16**	-0.845	0.300	**36**	2.735	0.939	**56**	6.315	0.998
	**17**	-0.666	0.339	**37**	2.914	0.949	**57**	6.494	0.998
	**18**	-0.487	0.381	**38**	3.093	0.957	**58**	6.673	0.999
	**19**	-0.308	0.424	**39**	3.272	0.963	**59**	6.852	0.999
	**20**	-0.129	0.468	**40**	3.451	0.969	**60**	7.031	0.999
**36**	**0**	-2.893	0.053						
	**1**	-2.714	0.062	**21**	0.866	0.704	**41**	4.446	0.988
	**2**	-2.535	0.073	**22**	1.045	0.740	**42**	4.625	0.990
	**3**	-2.356	0.087	**23**	1.224	0.773	**43**	4.804	0.992
	**4**	-2.177	0.102	**24**	1.403	0.803	**44**	4.983	0.993
	**5**	-1.998	0.119	**25**	1.582	0.829	**45**	5.162	0.994
	**6**	-1.819	0.140	**26**	1.761	0.853	**46**	5.341	0.995
	**7**	-1.640	0.162	**27**	1.940	0.874	**47**	5.520	0.996
	**8**	-1.461	0.188	**28**	2.119	0.893	**48**	5.699	0.997
	**9**	-1.282	0.217	**29**	2.298	0.909	**49**	5.878	0.997
	**10**	-1.103	0.249	**30**	2.477	0.923	**50**	6.057	0.998
	**11**	-0.924	0.284	**31**	2.656	0.934	**51**	6.236	0.998

Age (months)	TDAS Score	Z	Predicted Probability	TDAS Score	Z	Predicted Probability	TDAS Score	Z	Predicted Probability
	**12**	-0.745	0.322	**32**	2.835	0.945	**52**	6.415	0.998
	**13**	-0.566	0.362	**33**	3.014	0.953	**53**	6.594	0.999
	**14**	-0.387	0.404	**34**	3.193	0.961	**54**	6.773	0.999
	**15**	-0.208	0.448	**35**	3.372	0.967	**55**	6.952	0.999
	**16**	-0.029	0.493	**36**	3.551	0.972	**56**	7.131	0.999
	**17**	0.150	0.537	**37**	3.730	0.977	**57**	7.310	0.999
	**18**	0.329	0.582	**38**	3.909	0.980	**58**	7.489	0.999
	**19**	0.508	0.624	**39**	4.088	0.984	**59**	7.668	1.000
	**20**	0.687	0.665	**40**	4.267	0.986	**60**	7.847	1.000
48	**0**	-2.077	0.111						
	**1**	-1.898	0.130	**21**	1.682	0.843	**41**	5.262	0.995
	**2**	-1.719	0.152	**22**	1.861	0.865	**42**	5.441	0.996
	**3**	-1.540	0.177	**23**	2.040	0.885	**43**	5.620	0.996
	**4**	-1.361	0.204	**24**	2.219	0.902	**44**	5.799	0.997
	**5**	-1.182	0.235	**25**	2.398	0.917	**45**	5.978	0.997
	**6**	-1.003	0.268	**26**	2.577	0.929	**46**	6.157	0.998
	**7**	-0.824	0.305	**27**	2.756	0.940	**47**	6.336	0.998
	**8**	-0.645	0.344	**28**	2.935	0.950	**48**	6.515	0.999
	**9**	-0.466	0.386	**29**	3.114	0.957	**49**	6.694	0.999
	**10**	-0.287	0.429	**30**	3.293	0.964	**50**	6.873	0.999
	**11**	-0.108	0.473	**31**	3.472	0.970	**51**	7.052	0.999
	**12**	0.071	0.518	**32**	3.651	0.975	**52**	7.231	0.999
	**13**	0.250	0.562	**33**	3.830	0.979	**53**	7.410	0.999
	**14**	0.429	0.606	**34**	4.009	0.982	**54**	7.589	0.999
	**15**	0.608	0.647	**35**	4.188	0.985	**55**	7.768	1.000
	**16**	0.787	0.687	**36**	4.367	0.987	**56**	7.947	1.000
	**17**	0.966	0.724	**37**	4.546	0.990	**57**	8.126	1.000
	**18**	1.145	0.759	**38**	4.725	0.991	**58**	8.305	1.000

Age (months)	TDAS Score	Z	Predicted Probability	TDAS Score	Z	Predicted Probability	TDAS Score	Z	Predicted Probability
	**19**	1.324	0.790	**39**	4.904	0.993	**59**	8.484	1.000
	**20**	1.503	0.818	**40**	5.083	0.994	**60**	8.663	1.000

Z =-5.341+0.179(TDAS)+0.068(Age)

The formula to predict the probability of ASD based to these variables is defined as follows:

$$Pr(ASD) = e^{z}/(1+e^{z}) = e^{-5.341+0.179(TDAS)+0.068(Age)}/\left(1+e^{-5.341+0.179(TDAS)+0.068(Age)}\right),$$

where Pr(ASD) is the probability of ASD, TDAS is the TDAS score (ranged from 0–60), and Age is the age of the child suspected of having ASD (months).
